# Expansion of a paints and coatings solvent guide. Combining solvent guides and Hansen solubility parameters for greener solvent and solvent mixture selection

**DOI:** 10.1039/d6su00271d

**Published:** 2026-08-25

**Authors:** Erica Kemp, Harry Maslen, Helen F. Sneddon

**Affiliations:** a Green Chemistry Centre of Excellence, Department of Chemistry, University of York Heslington York YO10 5DD UK helen.sneddon@york.ac.uk

## Abstract

A substantive update to the 2024 paints and coatings solvents guide, scoring an additional 40 solvents, including those used as strippers, has been provided. The ranking of alternative solvents and solvent mixtures based on sustainability (or other) scores and Hansen Solvent Parameters (HSP) has been facilitated through modification of Albrecht and Strube's 2025 software. Two examples of scoring approaches are exemplified. Circle diagrams, showing a range of possible solvent mixtures as alternatives for a given solvent (or solvent mixture) have been developed and exemplified for two key solvents where there is particular interest in replacement, dichloromethane and *N*-methyl pyrrolidone. The nature of solvents and solvent mixtures required to map HSP space using both pharmaceutical and paints and coatings specific solvent lists has been plotted, highlighting under-served areas of solvent space for future research.

## Abstract 2

40 additional solvents are assessed against multiple sustainability criteria in a paints and coatings context. Hansen solvent parameters are used with different scoring criteria to generate lists of “greener” binary solvent mixtures for screening.

## Graphical abstract

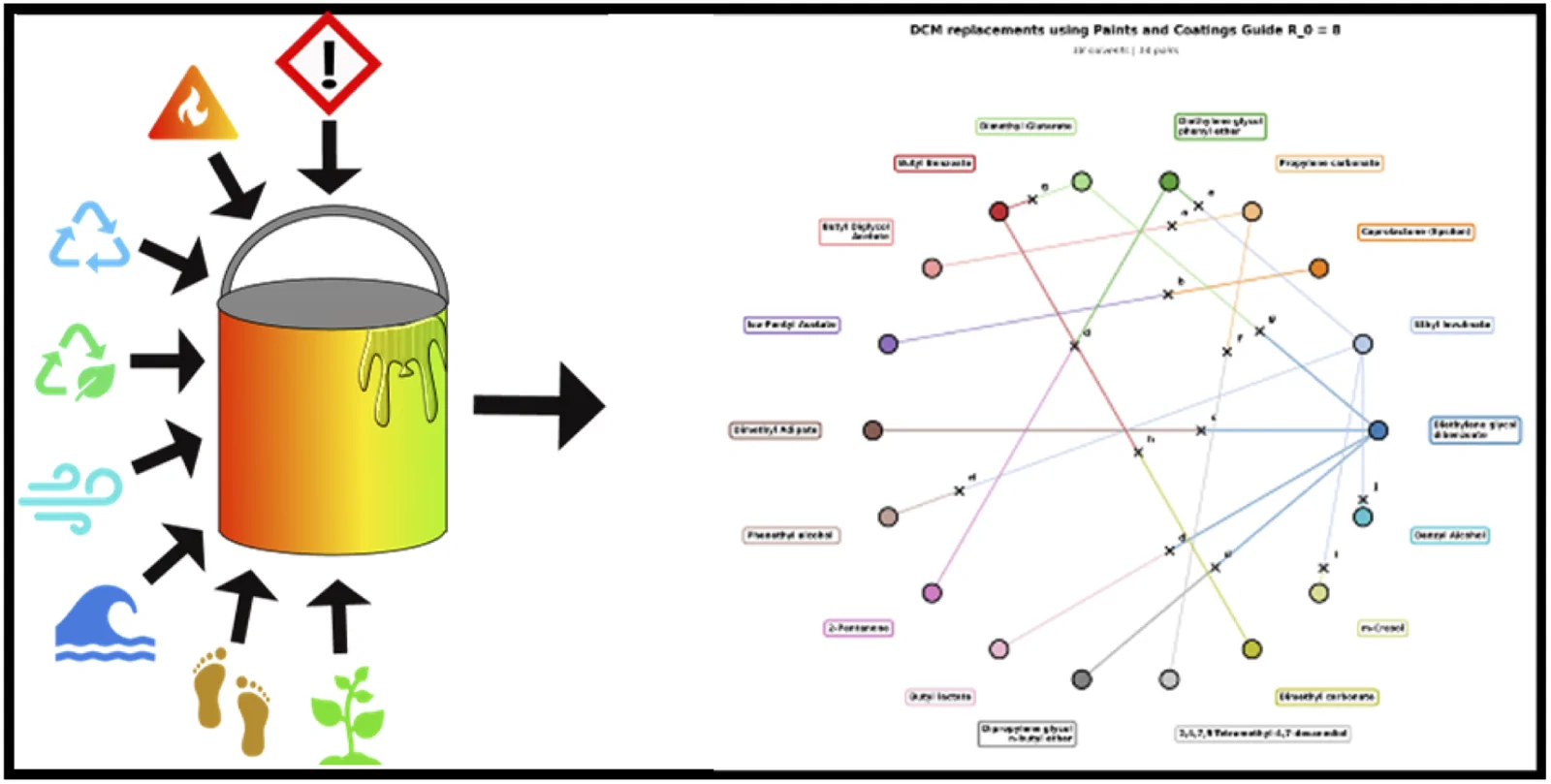

**Sustainability spotlight**

Solvents are critical to chemistry underpinning SDGs including Good Health and well-being (SDG 3), Industry Innovation and Infrastructure (SDG 9), and Responsible Consumption and Production (SDG 9). We here score 40 additional solvents in a paints and coatings context and facilitate the ranking of both pharmaceutical and paints and coatings solvents, based on a combination of Hansen solubility parameters and sustainability scores. Diagrams showcase a range of possible solvent mixtures as alternatives for a given solvent, exemplified for dichloromethane and *N*-methyl pyrrolidone. The nature of solvents and solvent mixtures required to map HSP space using both pharmaceutical and paints and coatings specific solvent lists has been plotted, highlighting under-served areas of solvent space for future research.

## 1. Introduction

Hansen Solubility Parameters (HSPs) have been used for nearly 60 years to understand solvents and issues of solubility, dispersion, diffusion, and chromatography.^1^ While HSPs alone cannot perfectly predict solubility, they can be used to identify solvents and solvent mixtures that are likely to be suitable and identify candidates for empirical screening or start points for formulation optimisation, and have achieved widespread use in solvent selection in the paintings and coatings sector,^2^ including as a tool for substituting fossil-based with biobased solvents^2,3^ Lists of “green” solvents have previously been provided for use in Hansen Solubility Parameter software,^4^ and in 2025 Albrecht and Strube published a Python tool for predicting optimal solvent blends based on Hansen solubility parameters.^5^

In recent years solvent guides, considering multiple facets of sustainability, have been a key tool in influencing the overall nature of the solvents used in industry. These have chiefly been applied in the discovery, development and ultimately manufacture of active pharmaceutical ingredients (APIs) in the pharmaceutical industry,^6–11^ or more recently analytical chemistry,^12^ and the original guides were designed with how solvents are typically used in the pharmaceutical industry in mind. The nature of the solvents scored, the relevance of the properties scored, and assumptions made about the conditions under which the solvents would be used, and how they might be disposed, do not necessarily translate to other use cases.

The GSK Solvent Selection Guide approach, considering multiple criteria, has been widely used with ∼1000 citations to date according to Scopus. Mindful that use of solvents by the paints and coatings industry has long dwarfed solvent use by the pharmaceutical sector, in 2024 we published a guide using analogous methodology specifically focussed on the nature and use of solvents in both industrial and consumer settings.^13^

We hereby expand on the 2024 paints and coatings solvent guide,^13^ scoring an additional 40 solvents, and specifically including solvents predominantly used as strippers as well as in paints and coatings formulations.

In addition, we elaborate on Albrecht and Strube's open access software,^5^ allowing the use of different lists of industry specific solvents, and context specific sustainability scores. The modified software enables a user to obtain a list of preferred solvents, or solvent mixtures, ranked in order of distance in Hansen solubility space from the desired solute, and/or by various sustainability credentials, as selected by the user, for screening. The size of the relative energy difference (RED), a dimensionless value used to indicate whether the solvent (or solvent mixture), and the interaction radius (*R*_0_), is within the solubility limits of the solute, can be customised. Typically, a RED value above 1 indicates a poor or non-solvent, while a value at or below 1 indicates a good or marginal solvent.

This software can therefore be used to find “greener” solvents or solvent mixtures suitable for solubilising a given substrate, or to find solvents, or solvent mixtures that might be suitable for screening as start points for replacing current solvents, where solubilising of solute is key to the application in question.

Mindful that dichloromethane (DCM) historically was used extensively in the paints and coatings industry, prior to EU legislation in 2010,^14^ and US legislation in 2014,^15^ and the use of dichloromethane in laboratory settings is increasingly restricted further to the introduction in 2024 in the US of methylene chloride regulation under the Toxic Substances Control Act,^15,16^ this approach has been used to generate lists of possible “greener” binary solvent mixtures for screening as alternatives to DCM.

Similarly, as *N*-methyl pyrrolidone (NMP) is restricted by REACH due to its classification as a reproductive toxicant and for its potential to cause serious eye, skin and respiratory irritation,^17^ this approach has been used to generate lists of possible “greener” binary solvent mixtures for screening as alternatives to NMP.

Iteration of this code across a hypothetical grid of HSP space additionally offers the intriguing possibility of mapping out which areas, *i.e.* which solutes, are most readily accessible through current “green” solvents and solvent mixtures for a particular application. Such an analysis has been done for different values of RED and *R*_0_ (see SI, S7–S10) and these plots suggest areas where future research is required for new “green” solvents and solvent mixtures.

## 2. Methodology

### 2.1 Solvent selection

The open access software provided by Albrecht and Strube^5^ is customisable to accept different solvent lists. Many solvent guides have been published over the years,^6,7,9–12,18^ however, for the purposes of this work, solvent lists for different applications were compiled as follows.

#### 2.1.1 Pharmaceutical industry relevant solvents

The 2016 iteration of the GSK Solvent Selection Guide^8^ provides a list of 154 solvents in the SI. In addition to solvents in common use across the industry, as explained in the SI of that publication, this list included several solvents that were at the time of publication being proposed as being of potential interest in this space but have not featured particularly in the literature in the intervening years (*e.g. N*,*N*-dimethyl octanamide, *N*,*N*-dimethyl decanamide, *N*,*N*-dimethylaniline) and several perfluorinated halogenated solvents (perfluorotoluene, perfluorocyclic ether, perfluorocyclohexane, perfluorohexane) now thought to be even less likely to be used as awareness of per- and polyfluoroalkyl substances (PFAS) issues has grown.^19^ Furthermore HSPs were not easily available (through use of Steven Abbott's website^20^ which lists the HSPs of >1000 solvents and the Accudyne website^21^) for all the solvents listed, and after consideration, this list was reduced to a list of 130 which was felt to reflect the more commonly encountered solvents.

#### 2.1.2 Solvents relevant to paints and coatings

The 77 solvents considered in the 2024 solvent sustainability guide for the paints and coatings industry^13^ were supplemented with an additional 40 solvents sourced from a combination of searching for solvents of relevance to paint strippers, and solvents listed by Albrecht and Strube, not previous considered in the 2024 guide. Whilst not all solvents were necessarily applicable to both consumer and industrial settings, for generation of one overarching paints and coatings guide, additional solvents were added to both the Consumer and Industrial versions (see SI). For solvents where differences in overall ranking were observed between the industrial and consumer guides the precautionary principle applied and the lower ranking was used throughout this combined paints and coatings work.

### 2.2 Solvent scoring

The methodology used in the 2024 paints and coatings solvent guide,^13^ was used to score the new 40 solvents. A summary of this methodology, and the differences between the consumer and industrial guides is provided in Table 1.

**Table 1:** Overview of scoring criteria for the paints and coatings guide

#	Scoring aspect	Rationale	Criteria used	Value range (score 1–10)	Consumer-specific guide	Industrial-specific guide	Notes
**Waste**							
1	Incineration	Solvents from paint manufacturing and disposal are frequently incinerated	NOx emissions (nitrogen-containing solvents penalised); enthalpy of combustion (ΔcH°, kJ g^−1^) considered as energy can be recovered; water solubility (wet solvents release less energy on combustion)	Score 1 (least “green”) – 10 (most “green”). Derived from geometric mean of emissions score and burn score	No water solubility factor; formula: √(emissions × burn)	Includes water solubility factor as there is a greater likelihood of energy recovery from solvent incineration, and potential contact with water in this process (smaller weighting than pharma guide). Formula: (emissions^2^ × burn^2^ × water)^(1/5)	
2	Recycling	Some industry processes allow solvent recovery and reuse; not applicable for consumer paints	Boiling point; reactivity; flammability and explosion potential; ease of drying; water solubility (potential for atmospheric moisture absorption during recycling)	Score 1–10. Derived from (BP^2^ × reactivity × flam/expl × ease of drying)^(1/5)	NOT included in consumer composite score. Consumer solvents are released to atmosphere on application and rarely collected for recycling (other than reuse projects)	INCLUDED in industrial composite score	Major methodology difference between the consumer and industry guides
3	Biotreatment	Solvents entering drains (*via* brush washing, spills) pass through wastewater treatment; nitrogen-rich or recalcitrant solvents place extra burden on biotreatment	Theoretical oxygen demand (ThOD, kJ kg^−1^)—penalises high-oxygen-demand solvents; nitrogen content (additional demand and ecological risk); water solubility—miscible solvents more likely to enter aqueous waste streams	Score 1–10 from geometric mean of ThOD score, *N*-content score, and aqueous potential score	Does NOT include a “release to air” component (removed *vs.* pharma guide): consumer paints intentionally release solvent to air on drying	Also does NOT include “release to air” component	
4	VOC emissions	Most solvents in paints are classified as VOCs; their tendency to evaporate affects air quality and regulatory compliance. Weighted more heavily than in the pharma guide given the evaporative role of paint solvents	Vapour pressure (mmHg at STP)—primary driver; boiling point—solvents with very low boiling points additionally penalised for acute high emission rates	Score 1–10 from geometric mean of vapour pressure score and boiling point score	Unchanged methodology. VOC score weighted more heavily in consumer composite formula (VOC^2^)	Unchanged methodology. VOC score weighted more heavily in industrial composite formula (VOC^2^)	Score reflects propensity to evaporate, not absolute regulatory VOC limits (which depend on amount, not type)

**Environment**							
5	Environmental impact – Aqueous	Solvents entering waterways (*via* runoff, drains, or spills) may harm aquatic organisms; bioaccumulation potential and biodegradability determine persistence of harm	Acute aquatic toxicity (LC_50_ fish, EC_50_ daphnia, IC_50_ algae—worst case, mg L^−1^); chronic toxicity (log KOW as bioaccumulation indicator; GHS H400–H413 phrases); biodegradability (BOD/ThOD ratio; ready biodegradability tests OECD 301; BOD data)	Score 1–10 from geometric mean of toxicity score, log KOW score, and biodegradability score	Methodology unchanged from pharma guide	Methodology unchanged from pharma guide	Log KOW used as chronic hazard indicator. GHS H phrases supplement toxicity data where test values unavailable
6	Environmental impact – Air	Evaporated solvents contribute to ozone formation and secondary indoor air pollutants; particularly important for consumer paints used indoors. Weighted more heavily than in the pharma guide	POCP (photochemical ozone creation potential, kg C_2_H_4_-eq/100 kg); SPCP (secondary product creation potential—modelled indoor air pollutant concentrations using INCHEM-Py/MCM); odour score (vapour pressure/odour threshold ratio)	Score 1–10 from geometric mean of POCP score, SPCP score, and odour score	SPCP ADDED for consumer guide. Calculated using INCHEM-Py v1.2 (MCM v3.3.1) simulating 10 ppb VOC in a residence (300 K, 45% RH, 0.76 h^−1^ air change). Captures indoor secondary pollutants: ONs, PANs, HCHO, O_3_, glyoxal, acetaldehyde	SPCP NOT used for industrial guide—higher ventilation requirements in factory environments mean indoor secondary product formation is less relevant	Air score weighted more heavily in both composite formulas (air × aqueous^2^)^(1/3) to reflect importance of VOC air impacts in paints context

**Health**							
7	Health hazard	Intrinsic toxicological hazard to humans from the solvent regardless of how much is inhaled or absorbed; includes reproductive toxicity, carcinogenicity, acute lethal toxicity, organ toxicity	OEL (occupational exposure limit, ppm)—initial benchmark from ACGIH TLV, NIOSH, OSHA, EU-OSHA, AIHA WEELs, UK WELs, German TRGS 900 (lowest value used); EU GHS H-phrase hazard classification applied *via* decision tree (H300/H310/H330 = score 1; H301/H311/H331/H314/H318 = score 2; H302/H312/H332/H373 = score 3; H315/H319/H335 = score 4)	Score 1–10 from the lower of OEL-derived score and GHS H-phrase score	Changed from pharma guide: if no OEL is available, score is based solely on GHS H-phrases. No presumed/default OEL value assigned	Changed from pharma guide: if no OEL is available, score is based solely on GHS H-phrases. No presumed/default OEL value assigned	Reproductive toxicity (H360/H362) or CMR classification triggers automatic score 1. Health score = √(health hazard × exposure potential)
8	Exposure potential	How readily a worker or consumer will actually be exposed *via* inhalation given typical use conditions; combines how volatile the solvent is with the risks of inhaling that solvent	OEL (minimum across all sources, ppm); relative rate of evaporation (RRE, relative to *n*-butyl acetate = 1.0); vapour hazard ratio (VHR = RRE/OEL)—the higher the VHR, the more rapidly a hazardous concentration can build up	Score 1–10 from VHR. High VHR = low score (more hazardous exposure potential)	Methodology unchanged from pharma guide	Methodology unchanged from pharma guide	VHR captures the combined effect of volatility and toxicity. A very toxic but non-volatile solvent may score better than a mildly toxic but highly volatile one

**Safety**							
9	Flammability & explosion potential	Solvents in paints represent significant fire and explosion risks during manufacture, storage, application, and disposal; particularly important given large-scale industrial use	Boiling point (°C); flash point (°C); auto-ignition temperature (°C); vapour pressure (mmHg); electrical conductivity (pS m^−1^—low conductivity = electrostatic ignition risk)	Score 1–10 from geometric mean of 5 component scores	Methodology unchanged	Methodology unchanged	Safety score = √(flammability & explosion × reactivity & stability). Pyrophoric/shock-sensitive hazards not scored as no qualifying solvents found in paints
10	Reactivity & stability	Some solvents can form peroxides on storage, self-react, decompose on heating, or react with acidic/basic paint components, presenting safety hazards	Peroxide formation potential (NOAA reactivity data: none/possible on concentration/forms readily); NFPA reactivity rating (0–3); acidity/basicity (pKa—very acidic or basic solvents penalised)	Score 1–10 from geometric mean of peroxide score, NFPA score, and acidity/basicity score	Special hazards component (pyrophoric, shock-sensitive) NOT included—none of the assessed paints solvents meet this criterion	Special hazards component NOT included for same reason – none of the assessed paints solvents meet this criterion	THF scores particularly poorly due to peroxide formation potential (score 1). Score reflects self-reactivity; reaction with paint components handled separately through formulation assessment

**Sourcing**							
11	Global warming potential (GWP)	New for paints guide. Reflects the carbon footprint of manufacturing the solvent. Full LCA excluded as data unavailable for many solvents	Cradle-to-gate GWP (kg CO_2_ eq. per kg solvent) from CCalc 2 and literature. Where unavailable, near-neighbour estimation used based on molecular complexity and molar mass. Divided into quartiles: Q1 (lowest GWP) = score 4; Q2 = score 3; Q3 = score 2; Q4 (highest GWP) = score 1	Score 1–10 from quartile rank. *Q*1 = 10 (greenest), *Q*4 = 1 (least green)	New for this guide. Methodology identical for both use cases	New for this guide. Methodology identical for both use cases	Sourcing score = √(GWP × biobased). Sourcing was excluded entirely from the pharma guide (LCA not included in composite). Major addition in paints guide
12	Biobased potential	New for paints guide. Reflects the potential for the solvent to be sourced from renewable feedstocks; responds to industry net-zero targets and growing interest in biobased materials	Qualitative assessment of commercial biobased availability: Score 4 = current commercial whole-structure biobased source; score 3 = potential commercial biobased source (unconfirmed); score 2.5 = partially biobased (at least one component from bio); score 2 = potential future commercial biobased source; score 1 = no likely biobased source	Score 1–10 derived directly from biobased rating (×2.5 to give 1–10 scale). Comments in SI excel files provide details of specific biobased routes considered	New for this guide. Methodology identical for both use cases	New for this guide. Methodology identical for both use cases	Inherently crude and subject to change as new routes emerge. Does not assess whether biobased route is sustainable (land use, agricultural waste *vs.* food crop). TRL and commercial scale not factored in

Overall traffic light scores of “red” (most issues), “amber” (some issues) and “green” (few issues) have been applied consistent with the methodologies published in the respective papers.^8,13^

However, the parameters can be adjusted to reflect other specific sub-category scores as might be appropriate to a particular application, for example biobased content, or VOC emissions. Factors not directly related to sustainability could also be ranked in a similar way, for example, melting or boiling point, or, should users have the data applicable to their situation available, cost.

### 2.3 Solvent shortlisting for screening

Any proposed scoring approach must be treated with caution. A solvent mixture cannot in general be assumed to inherit its hazard or sustainability profile directly from its single components. There are many documented examples of, for example, minimum flash points of mixtures being lower than either of the composite solvents.^22–25^ In addition toxicity can also be related to co-solvent effects, and in the case of dermal absorption, mixtures can significantly enhance skin permeation relative to the neat compounds,^26,27^ including with an *N*-methyl pyrrolidone mixture.^28^

In occupational exposure assessment, additive models are commonly applied when multiple substances produce similar effects, such as respiratory irritation or Central Nervous System (CNS) depression. For example, Occupational Safety and Health Administration (OSHA) explicitly uses an equivalent exposure approach based on the sum of concentration-to-limit ratios for mixtures of contaminants with similar effects.^29^ Classification, Labelling and Packaging (CLP) regulation also dictates that the assignment of mixtures for multiple hazard classes – several of which are particularly relevant to paint and coating solvents – be concentration-driven and work additively.^30^ This approach generates conservative safety assessments for solvent mixtures which may not be fully captured by a worst component approach.

However, we believe there is value in applying some simple over-arching triaging of solvent mixtures by sustainability or other criteria in cases where full assessment of all solvent mixtures is unlikely to be feasible. We believe, for example, that a “worst component” approach may be helpful to apply simplistic composite scores to large numbers of hypothetical mixtures of solvents in order to winnow down prospective candidates for screening. Such an approach would later require a detailed analysis of the specific physicochemical and toxicological properties of any promising mixtures to be rigorously assessed, accounting for the additivity (or more complex interaction) of hazards.

Similarly, a weighted average of scores might be more appropriate for some criteria, again on the understanding that this approach is proposed to exist alongside full risk assessment, and that more rigorous assessment of promising mixtures would be required before scale up, or further commitment of time, or resources.

#### 2.3.1 Scoring based on lowest scoring component

The code has been modified such that the output csv file of solvents and solvent mixtures listed in order of RED from the specified solute (or solutes, as the code can handle up to 5 solutes or polymers) is now annotated with (and filterable by) a red (R), amber (A), or green (G) label, as applicable to the worst ranked solvent present in a mixture. Whilst clearly not always a sufficiently rigorous approach for handling mixtures, this was felt in certain scenarios to be an appropriate way to take into account that not all hazards scale with volume used, and this approach, or one analogous to it, is considered justified when specific classes of solvent need to be entirely avoided.

#### 2.3.2 Scoring based on composition dependent weighting

The code has also been modified such that a composition dependent weighting can be given to numerical scores (or sub-scores). As described above, this approach will also not always be an appropriate way to handle mixtures, however we exemplify it for biobased-content score, where it could have use in triaging out mixtures with particularly low biobased content, and – if accurate, source specific, data were available for a subset of solvents – the approach could be trivially extended to allow for focus only on mixtures of greater than a given percentage biobased content predicted to be worth screening to solubilise a given substrate of interest.

Additive approaches, multicomponent optimisation, or triaging through various iterations of different scoring systems might also be envisaged depending on specific desired criteria for a given application.

### 2.4 Mapping of coverage of Hansen solvent parameter space

Iteration of this code across a grid of HSP space, separating each point by 0.5, was used to give visualisations of what might be anticipated to be accessible from blends of red, amber and green solvents, using either the pharmaceutical solvents or combined paints and coatings solvent lists for various values of RED and *R*_0_. Percentage coverage could be quoted for each “cloud” of space. The code, modified from Albrecht and Strube's start point, is made available in the SI.

## 3. Results

### 3.1 The updated paints and coatings guide

Forty solvents (18 of which overlap with the pharmaceutical solvents list) were scored according to the consumer (Fig. 1) and industrial (Fig. 2) paints and coatings heuristics^13^ for:

**Fig. 1:**
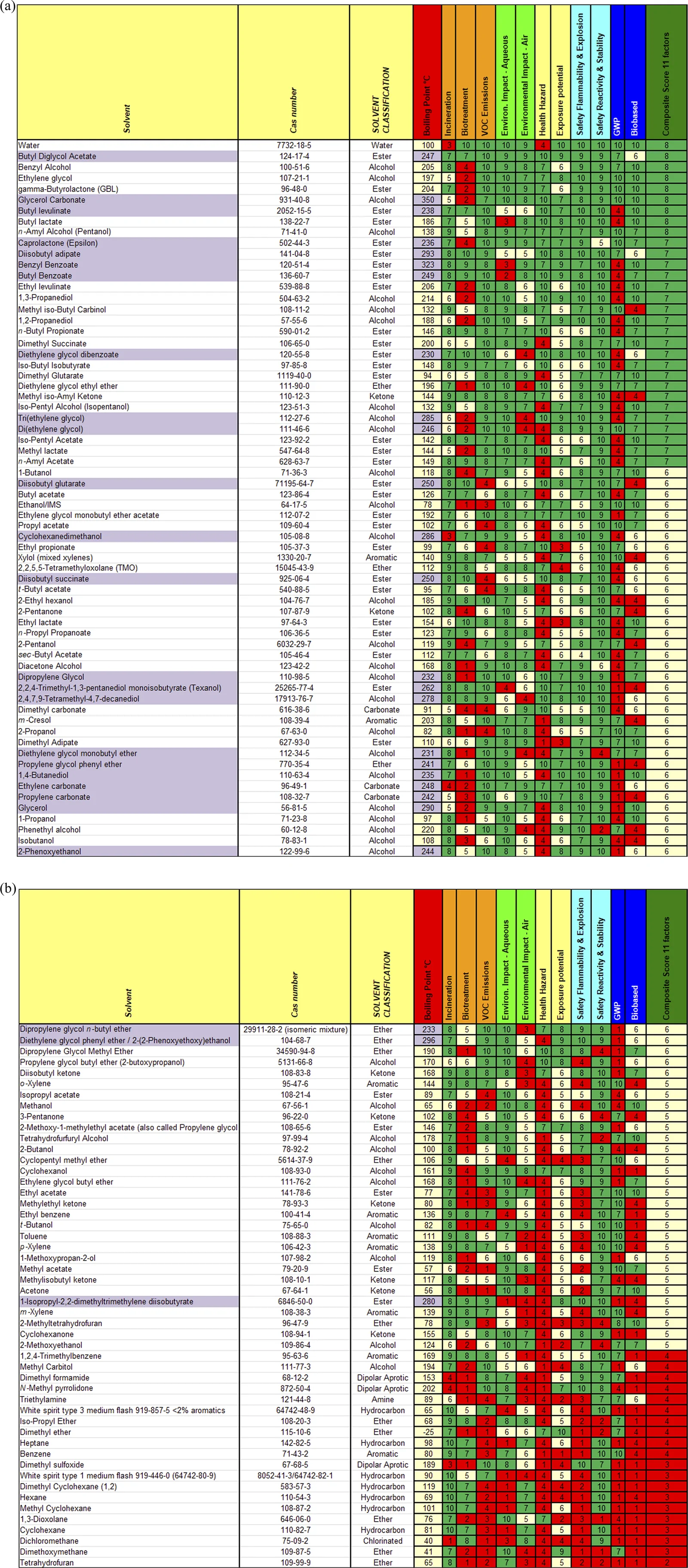
(a and b) Updated single page version of the guide for paints and coatings, applying the consumer scoring. Solvents highlighted in purple have boiling points >230 °C, which identifies them as existing or potential coalescent solvents in waterborne paints.

**Fig. 2:**
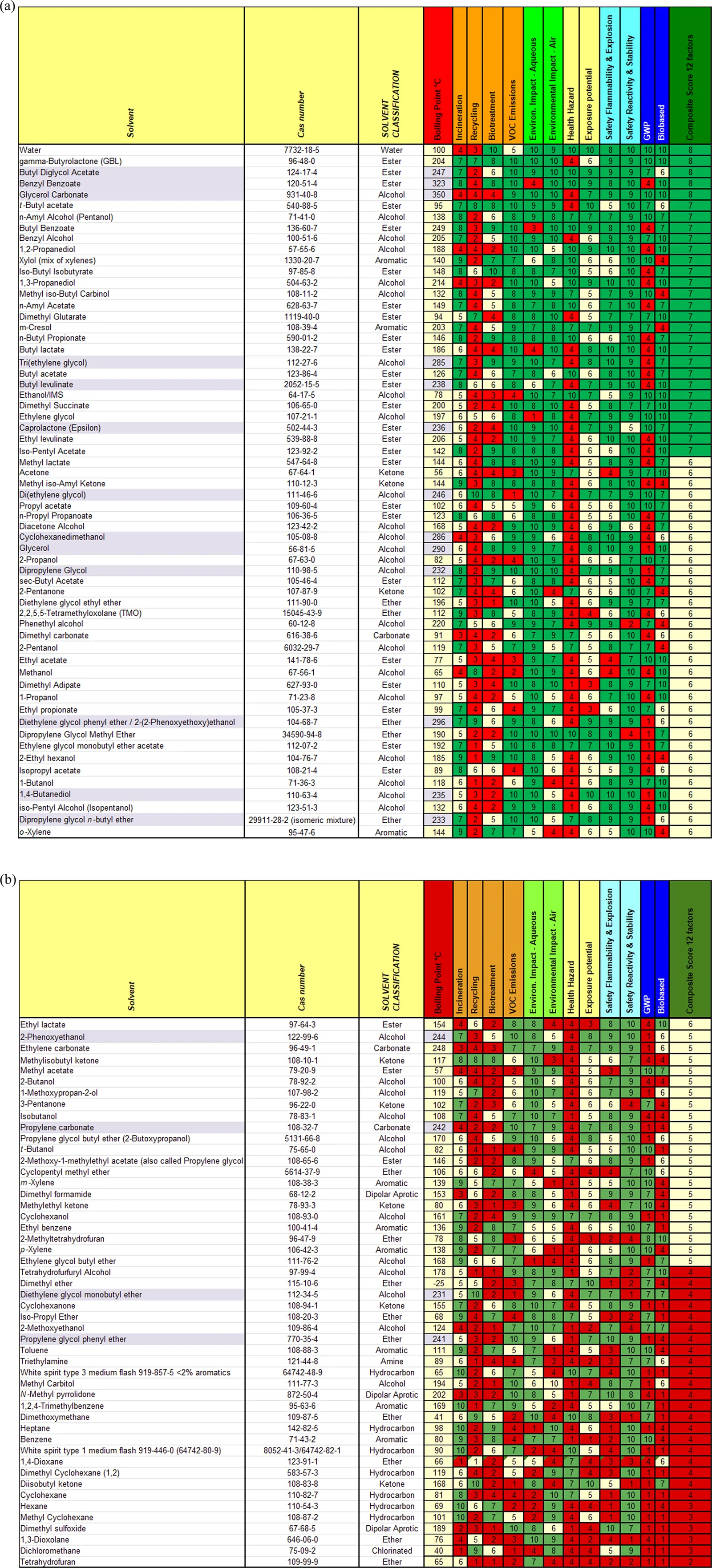
(a and b) Updated single page version of the industrial solvent selection guide for paints and coatings. Solvents highlighted in purple have boiling points >230 °C, which identifies them as existing or potential coalescent solvents in waterborne paints.

1. Consumer decorative paints – paints designed to be applied by the general public in home environments. Whilst consumer paints are now predominantly waterborne, some trim paints and primers are still solvent borne. Drying takes place at ambient temperature.

2. Air-drying industrial paints – paints designed to be applied by professional applicators in factory environments with the protection of local exhaust ventilation and emissions capture. Drying takes place at ambient or slightly elevated temperature.

Differences between the scoring approaches are summarised in Table 1 and provided in more detail in the 2024 paints and coatings guide.^13^ The 40 solvents newly scored here are: benzyl benzoate, butyl benzoate, 1,2-dimethyl cyclohexane, hexane, methyl cyclohexane, heptane, cyclohexane, xylene, *iso*-butyl *iso*-butyrate, *n*-butyl propionate, *n*-amyl acetate, *iso*-pentyl acetate, *m*-cresol, methyl *iso*-amyl ketone, *n*-propyl propanoate, *sec*-butyl acetate, *iso*-propyl ether, methyl *iso*-butyl carbinol, *n*-amyl alcohol (pentanol), *iso*-pentyl alcohol (isopentanol), butyl diglycol acetate, cyclohexanol, benzyl alcohol, caprolactone (epsilon), diacetone alcohol, 1,3-dioxolane, dipropylene glycol, dipropylene glycol methyl ether, gamma-butyrolactone (GBL), glycerol carbonate, methyl carbitol, tetrahydrofurfuryl alcohol, dichloromethane, dimethoxymethane, dimethyl ester, phenethyl alcohol, dimethyl sulfoxide, dimethyl adipate, dimethyl succinate.

### 3.2 Visualisation of the pure solvents in HSP space

The spread of solvents from the pharmaceutical (Fig. 3) and paints and coatings (Fig. 4) guides can be seen in HSP space. Each solvent has been coloured according to its overall traffic light (RAG) scores of “red” (most issues), “amber” (some issues) and “green” (few issues) and these scores have been applied consistent with the methodologies published in the respective papers, with for the paints and coatings guide whichever is the lowest rank used in instances where there are differences between the consumer and industrial guides. (See SI S1 and S2).^8,13^ As might be expected for the greater range of purposes to which the pharmaceutical industry relevant solvents are typically used, the coverage is wider in Fig. 3. The absence of “green” solvents in the non-polar, low *δ*_P_ (polar) region across both classes of solvents is consistent with previous observations.^13,31,32^

**Fig. 3:**
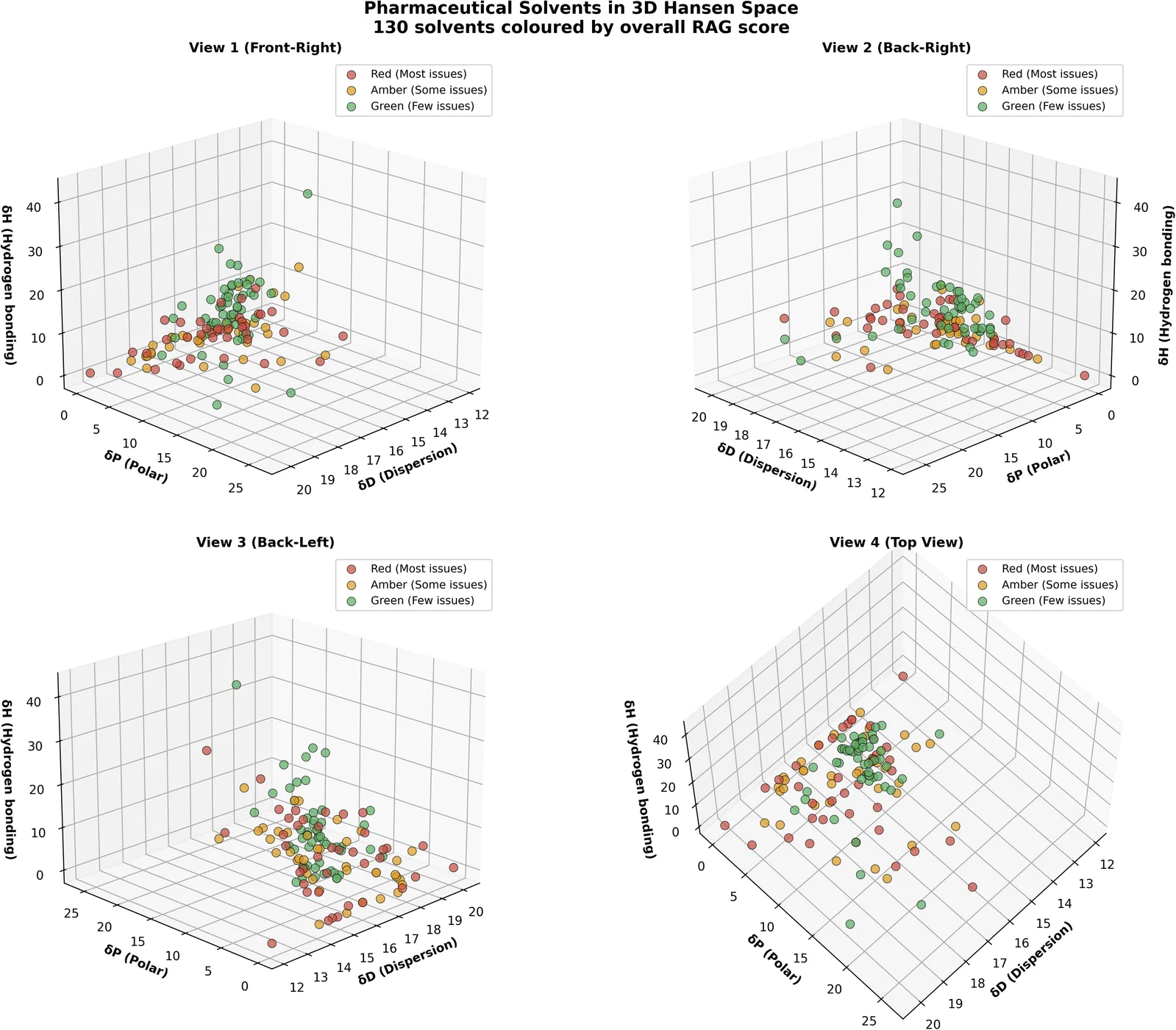
Visualisation of the pharmaceutical guide solvents in HSP space.

**Fig. 4:**
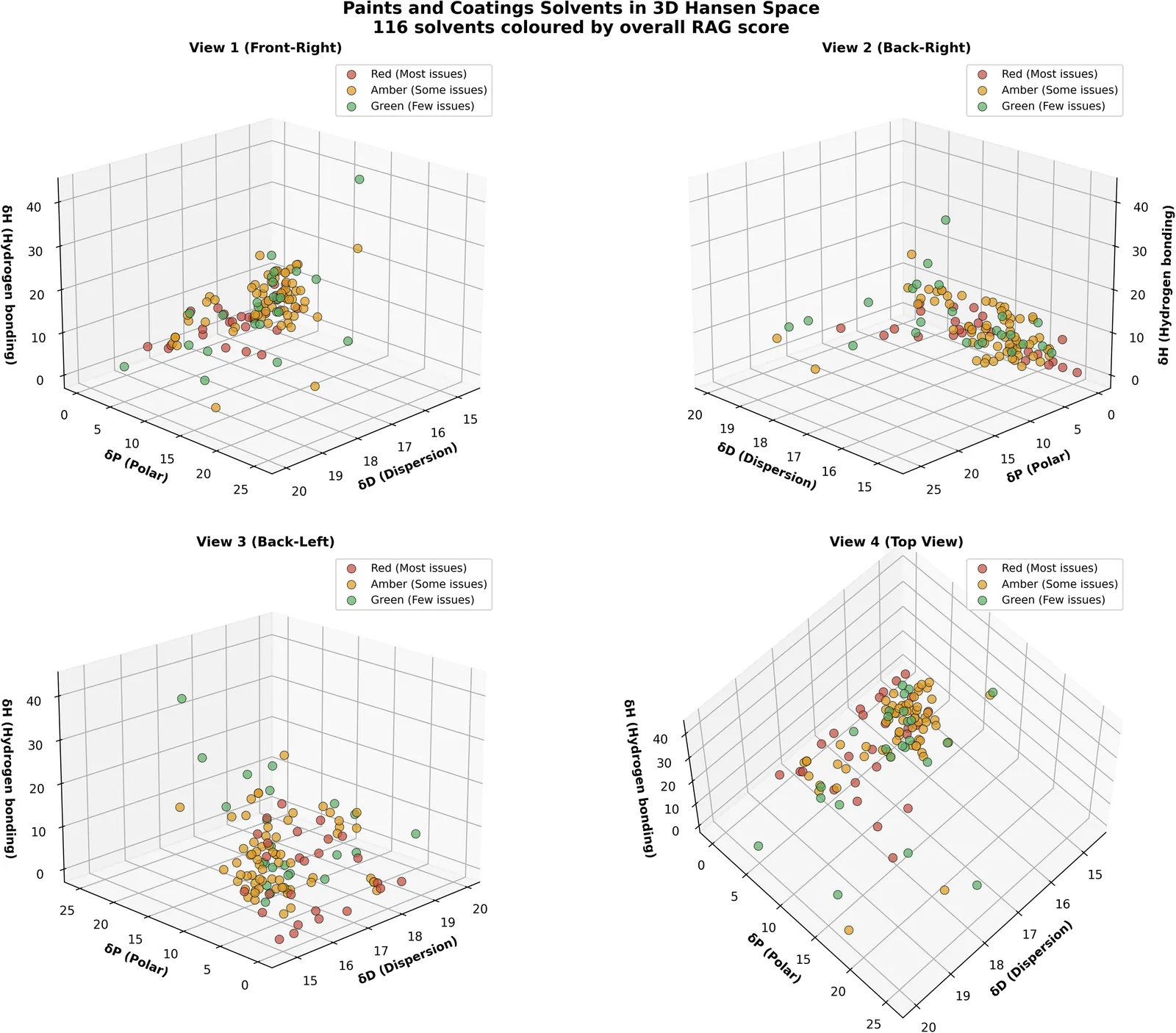
Visualisation of the paints and coatings guide solvents in HSP space.

### 3.3 Suggestion of possible alternative solvents and solvent mixtures

The tool can be used, with various values of *R*_0_ and RED, to explore the solvents or solvent mixtures likely to solubilise a particular substrate of known HSPs. Alternatively, whilst both dichloromethane and *N*-methyl pyrrolidone are known for solubilising a wide range of substrates, as a demonstration of how this tool can be used to rank solvent mixtures for screening solubility, one could consider how solvent mixtures might be explored as alternatives for solvents where usage is being discouraged or phased out.

For both the pharmaceutical and paints and coatings solvents lists, looking at step sizes of solvent ratios of 5% (chosen as a practical compromise as a readily computed, simple to work with, level of granularity, other step sizes can be selected, by adjusting the excel parameter set up file), or a given *R*_0_ value (here we have chosen *R*_0_ = 8 as typical of the distance of a marginal solvent from a polymer.^33^) lists can be generated, and filtered so any given solvent pair only returns the ratio of solvents with the lowest RED, to give lists of possible “green” and “amber” alternative solvent mixtures (see SI S3–S6).

#### 3.3.1 Alternatives to dichloromethane

Dichloromethane historically was used extensively in the paints and coatings industry, prior to EU legislation in 2010,^14^ and US legislation in 2014,^15^ and the use of dichloromethane in laboratory settings is increasingly restricted further to the introduction in 2024 in the US of methylene chloride regulation under the Toxic Substances Control Act.^16^

Whilst the lists generated by this code (see SI S3 and S5) are anticipated to be useful, they do not immediately highlight which solvents are appearing most frequently with other partners as possible replacements.

A visualisation was therefore developed (code available in the SI) to show solvent mixtures for the top X (number can be specified by user) solvents giving the lowest RED values for a particular scenario (solvent % step sizes, *R*_0_, HSP for solute/solvent seeking replacement, filtered to show “green” and “amber” or just “green” solvents). The visualisations depict the ratio of the solvent mixtures by means of a cross on the line between two solvents, and have a legend showing the RED value. The specifics of such diagrams will vary depending on the parameters chosen but examples are shown for possible binary mixtures of Green and Amber solvents from the pharmaceutical solvents list (Fig. 5) and paints and coatings solvent list (Fig. 6) that might mimic dichloromethane, with an *R*_0_ of 8 (Full tables available in the SI S3 and S5).

**Fig. 5:**
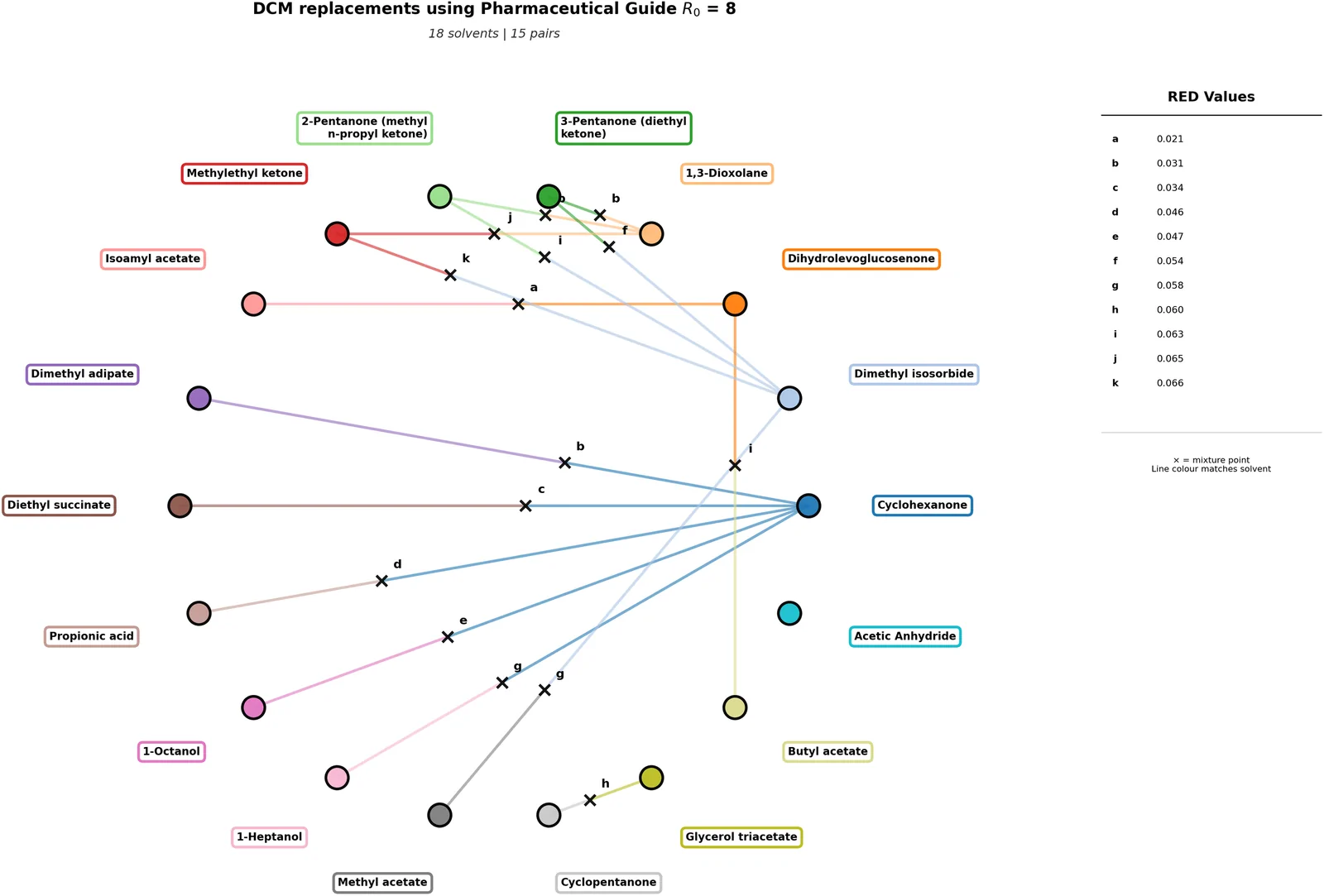
Selected suggested “green” and “amber” binary solvent mixture alternatives to dichloromethane using the pharmaceutical solvents list. Colours included for disambiguation of one solvent from another. Not indicative of any scoring system.

**Fig. 6:**
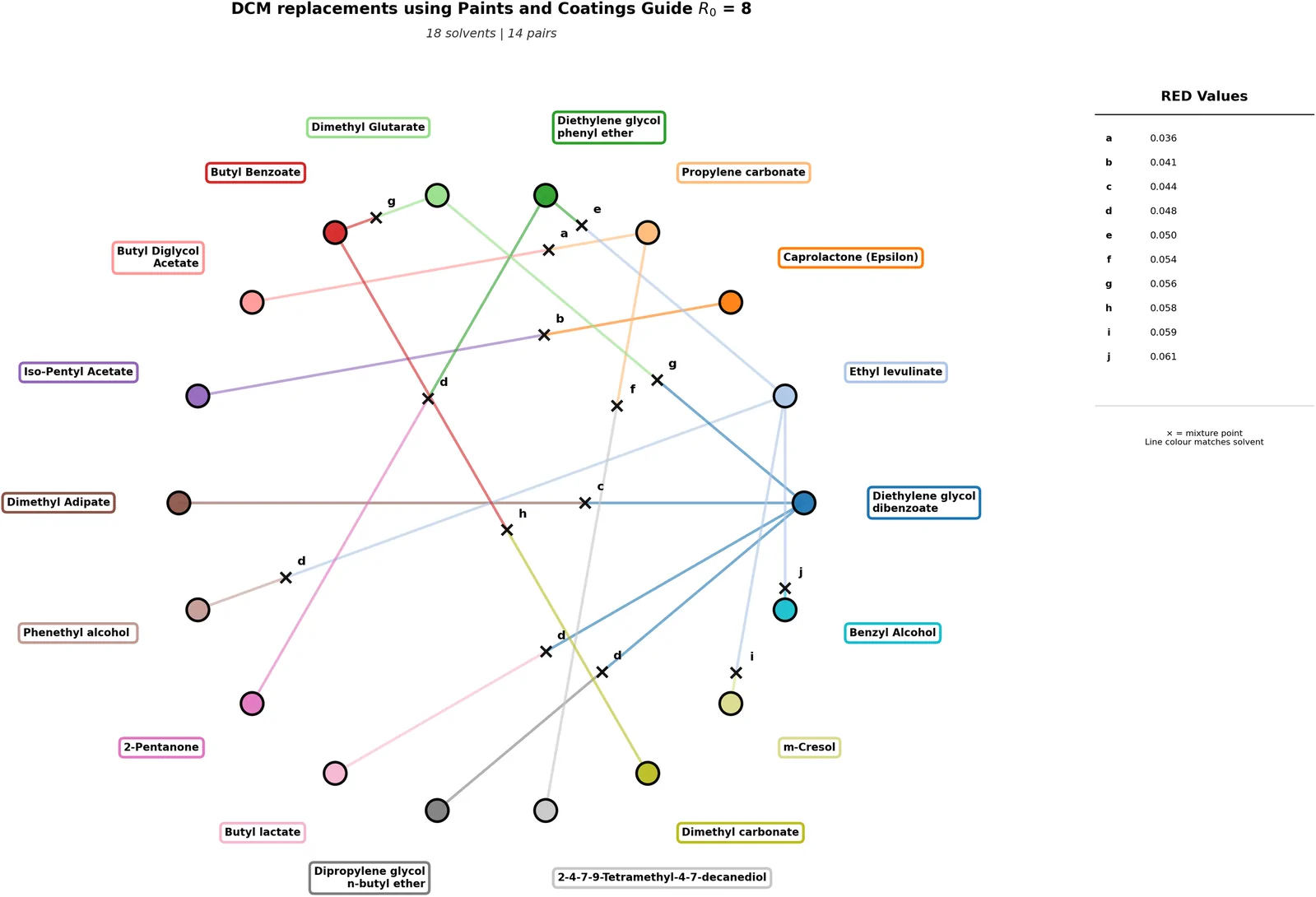
Selected suggested “green” and “amber” binary solvent mixture alternatives to dichloromethane using the paints and coatings solvents list. Colours included for disambiguation of one solvent from another. Not indicative of any scoring system.

#### 3.3.2 Alternatives to *N*-methyl pyrrolidone

*N*-methyl pyrrolidone (NMP) is restricted by REACH due to its classification as a reproductive toxicant and for its potential to cause serious eye, skin and respiratory irritation.^17^ As such there is interest in binary mixtures than can mimic the Hansen solubility parameters of *N*-methyl pyrrolidone, and lists have been generated from both solvent lists (see SI S4 and S6). The solvent mixtures can be visualised as a diagram considering possible binary mixtures of green and amber solvents from the pharmaceutical solvents list (Fig. 7) and paints and coatings solvent list (Fig. 8) that might mimic *N*-methyl pyrrolidone, within an *R*_0_ of 8, chosen as a typical example.

**Fig. 7:**
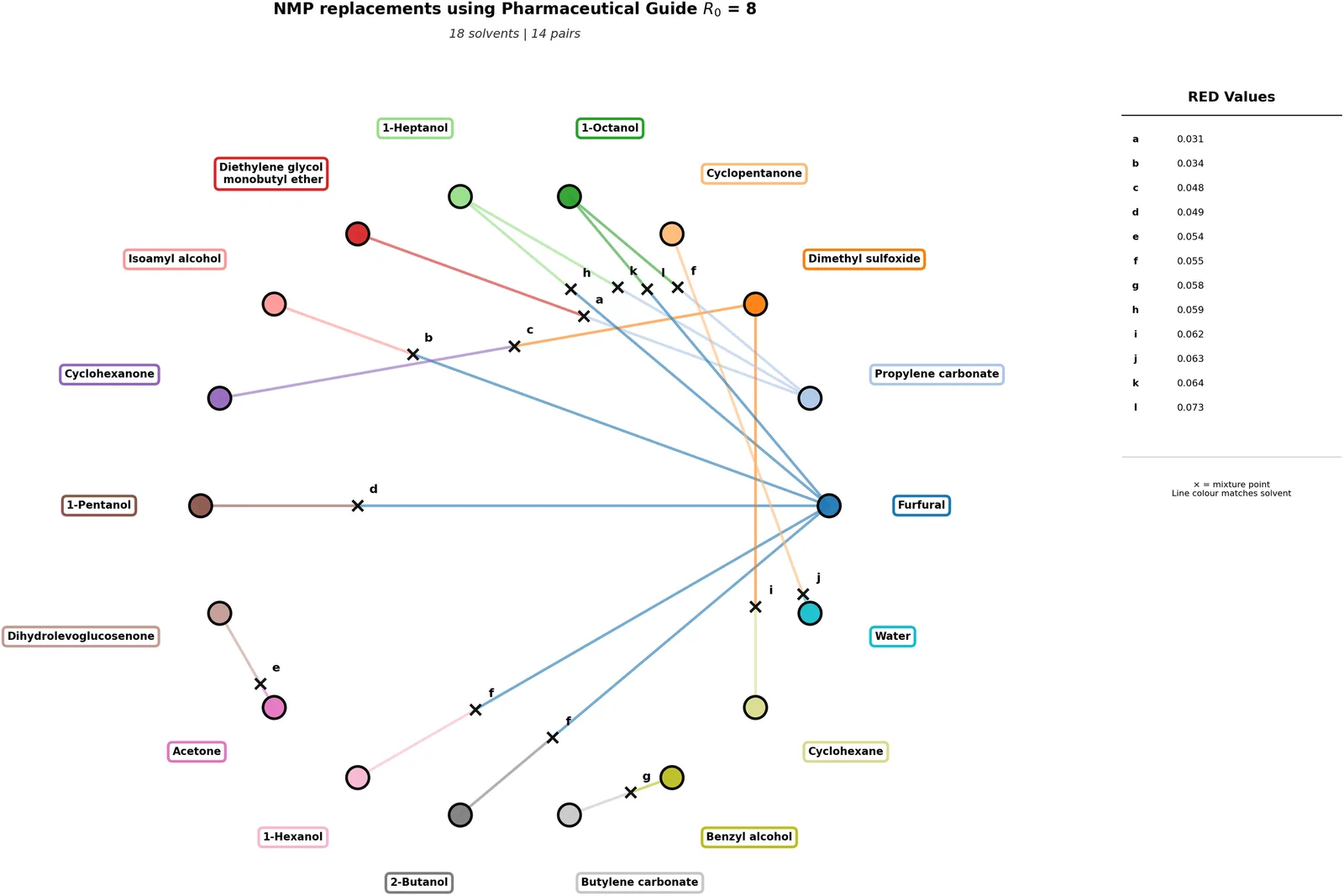
Selected suggested “green” and “amber” binary solvent mixture alternatives to *N*-methyl pyrrolidone using the pharmaceutical solvents list. Colours included for disambiguation of one solvent from another. Not indicative of any scoring system.

**Fig. 8:**
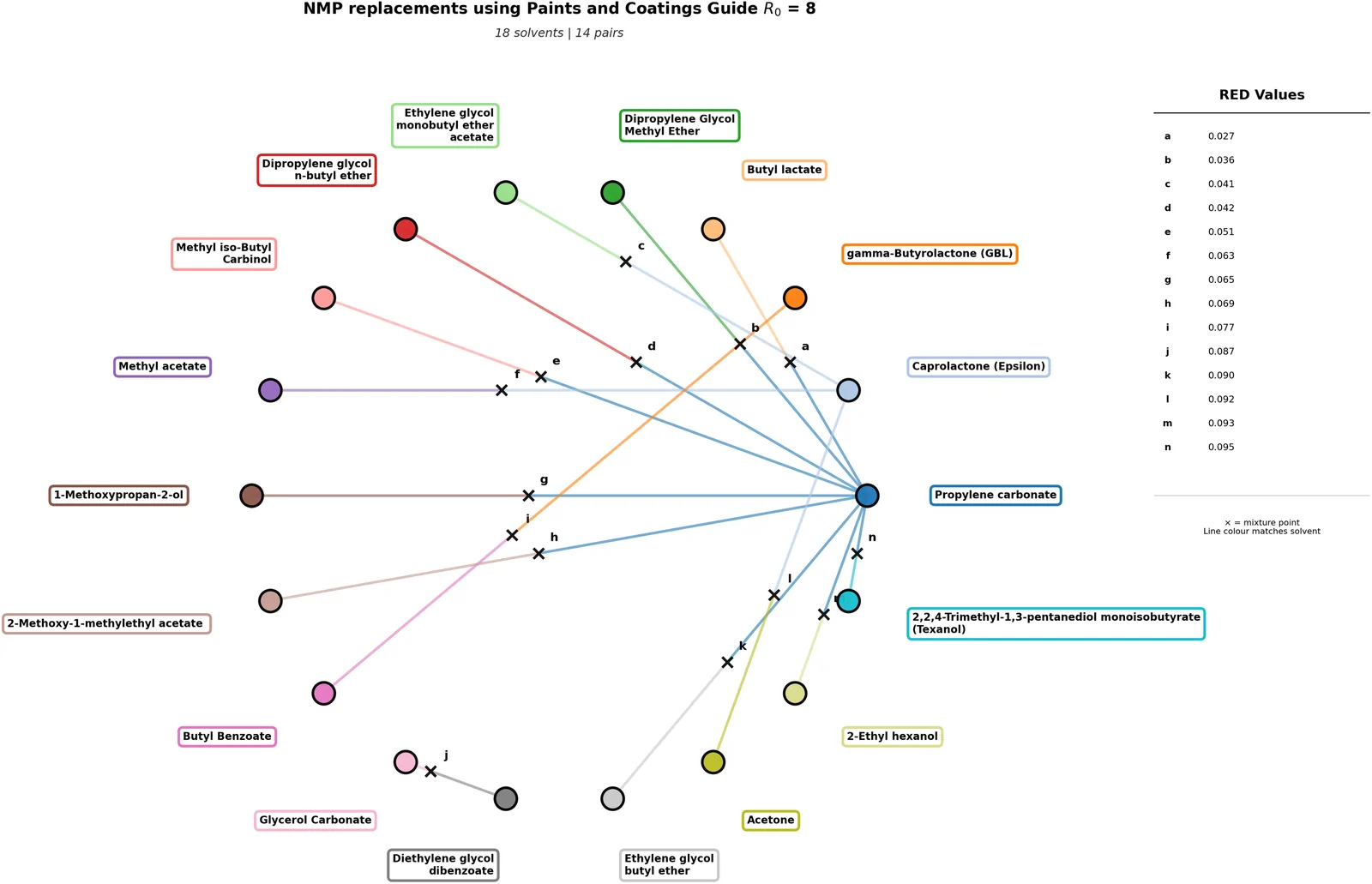
Selected suggested “green” and “amber” binary solvent mixture alternatives to *N*-methyl pyrrolidone using the paints and coatings solvents list. Colours included for disambiguation of one solvent from another. Not indicative of any scoring system.

Furthermore, a diagram is also presented in which the red, amber green overall classifications were not used (and indeed one of the solvents suggested, 1,4-dioxane, scores overall “red”), and scoring only considered biobased (or potentially biobased) content (Fig. 9). In this case greater weighting was given to solvent mixtures with a weighted biobased score >4. This is to exemplify how composition dependent weighting can be used to produce tailored or bespoke lists of binary mixtures to address specific sustainability criteria.

**Fig. 9:**
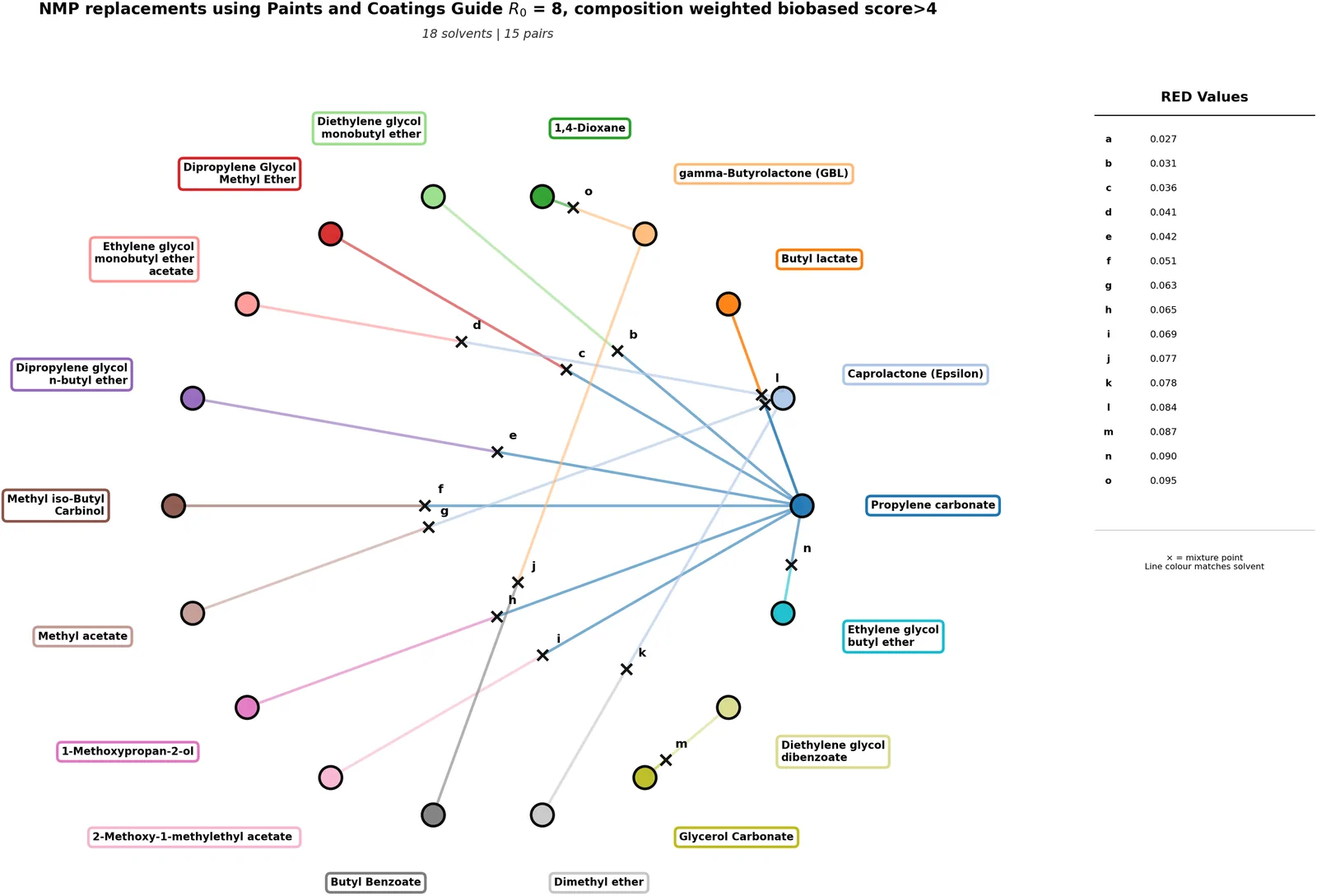
Example of using composition weighted average scoring. Suggested binary solvent mixture alternatives to NMP using the paints and coatings list with an overall biobased score of greater than 4. Colours included for disambiguation of one solvent from another. Not indicative of any scoring system.

### 3.4 Mapping of accessible space using the pharmaceutical solvents list

The code was iterated across a hypothetical grid of HSP space separating each point by 0.5, considering solvent mixtures refined to 5%, and with an (arbitrarily chosen) RED value of 0.3, and *R*_0_ of 8 (additional plots available in SI show how these visualisations and statistics can vary with different RED and *R*_0_ values) to give visualisations of what might be anticipated to be accessible HSP space from blends of red, amber and green solvents, using the pharmaceutical solvents list (Fig. 10). The Red, Amber, Green, (RAG) score for each point is determined in this visualisation by the worst ranked solvent present in the mixture, irrespective of composition.

**Fig. 10:**
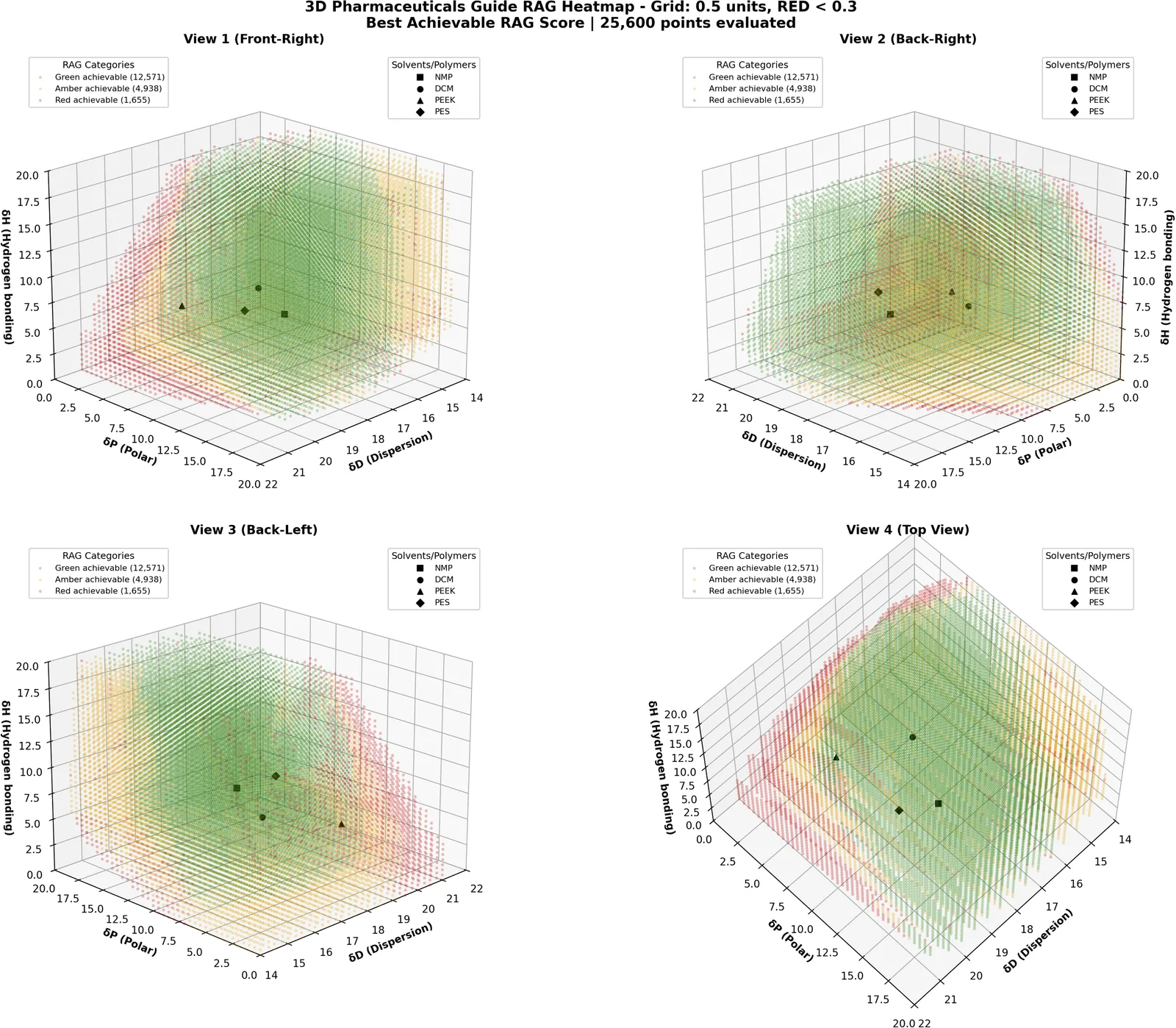
Map of the best achievable RAG score for solvents or solvent mixtures likely to solubilise a given point in HSP space using 130 pharmaceutically relevant solvents. Green points indicate that both solvents in the binary mixture have a RAG score of green, amber points indicate that one or both of the solvents have a RAG score of amber, and red points indicate that one or both of the solvents have a RAG score of red.

Of 25 600 points across this grid, 19 164 grid points were deemed to have achievable solutions (RED < 0.3) with this list of 130 solvents (74.9% coverage of this space).

Of the area deemed to have an achievable solution, “green” solvents or solvent mixtures could be used for 12 571 grid points (49.1% of the total space, or 65.6% of the “achievable cloud”). “Amber” solvents or solvent mixtures could be used for 4938 grid points (19.3% of the total space or 25.8% of the “achievable cloud”). “Red” solvents were required for 1655 grid points (6.5% of the total space or 8.6% of the “achievable cloud”).

### 3.5 Mapping of accessible space using the paints and coatings solvents list

The code was iterated across a hypothetical grid of HSP space separating each point by 0.5, considering solvent mixtures refined to 5%, and with an RED value of 0.3 (additional plots available in SI) to give visualisations of what might be anticipated to be accessible HSP space from blends of red, amber and green solvents, using the paints and coatings solvents lists, Fig. 11.

**Fig. 11:**
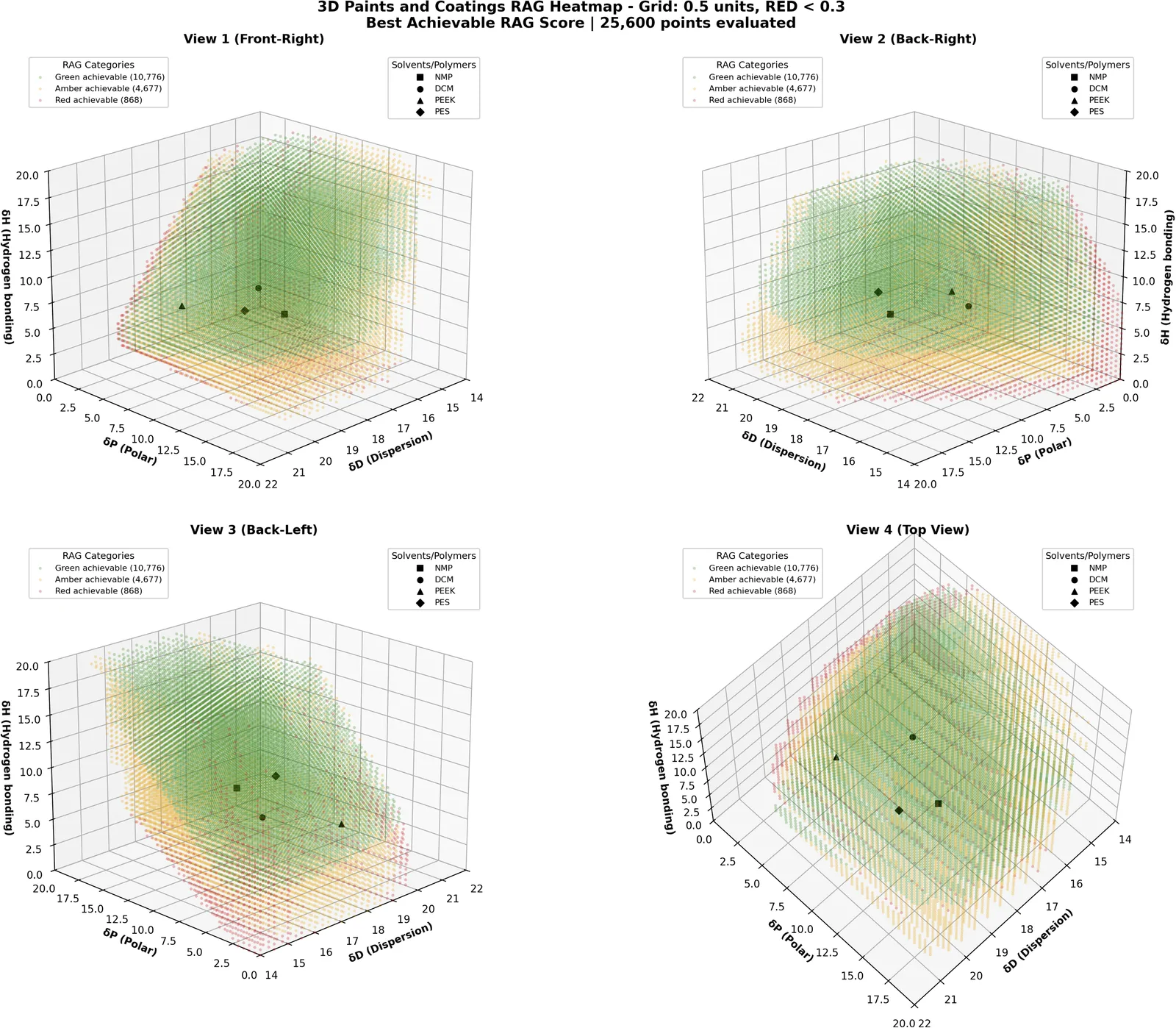
Map of the best achievable RAG score for solvents or solvent mixtures likely to solubilise a given point in HSP space using 116 paints and coatings solvents. Green points indicate that both solvents in the binary mixture have a RAG score of green, amber points indicate that one or both of the solvents have a RAG score of amber, and red points indicate that one or both of the solvents have a RAG score of red.

Of 25 600 points across this grid, 18 391 grid points were deemed to have achievable solutions (RED < 0.3) with this list of 116 solvents (71.8% coverage of this space).

Of the area deemed to have an achievable solution, “green” solvents or solvent mixtures could be used for 13 142 grid points (51.3% of the total space, or 71.5% of the “achievable cloud”). “Amber” solvents or solvent mixtures could be used for 4618 grid points (18.0% of the total space or 25.1% of the “achievable cloud”). “Red” solvents were required for 631 grid points (2.5% of the total space or 3.4% of the “achievable cloud”).

## 4. Discussion

### 4.1 Caveats

#### 4.1.1 Utility

The additional 40 solvents had all been listed (either by Albrecht and Strube^5^ or in our findings) as being of interest to the paints and coatings industry, and were added to the consumer and industrial guide as suggested by literature, or to both in order to score using both assessments. Presence of a solvent in one list or the other or both should not therefore be taken as definitively implying that such solvents are being, or should be, used in such scenarios.

The solvent lists and diagrams generated are purely demonstrative, for proposed use generating shortlists for screening. Whilst the HSP approach underpinning this work is well validated in the literature,^34,35^ with numerous practical case studies where this has been applied in the paints and coatings space,^3^ no claims are made that any of the suggested alternatives will work as drop-in replacements for any specific application.

We would urge caution, and Fig. 10 and 11 show, in addition to the position of NMP and DCM in the “clouds” of space accessible by “green” binary mixtures, two exemplar polymers: polyethersulfone (PES) and polyether ether ketone (PEEK). The location of both points suggests that (under the given RED values and *R*_0_ values) “green” binary solvent suggestions will be suggested. Indeed this is the case (see SI S11). However, the literature suggests both polymers have proved challenging to fabricate using greener solvents with PES having literature support for more promising options,^36–40^ (including some from these lists) than PEEK, notably toward the very outer edge of the “green” solvents cloud.^41^

Different lists will be obtained if different *R*_0_ values are used, or if the solvents are constrained in being mixed at finer, or coarser, gradations than 5%. Users are invited to customise as to their requirements. Work to date has so far only considered binary mixtures. Extension to ternary mixtures is possible, but likely to involve additional barriers in the practical use of such mixtures.

In addition to there being no guarantee that these solvent mixtures will solubilise the required substrates as predicted, there are many applications for which other criteria (functional group compatibility, melting points, boiling points, viscosity, necessity of using a single solvent, transparency at a given wavelength for monitoring of a reaction, *etc.*) would additionally need to be considered. Several of the solvents in these lists will have reactivities incompatible with many solvent uses. The chemist using this tool will be required to filter according to additional requirements specific for their purpose, however, such lists might, in some circumstances provide a start point for screening of alternative solvents, or the development of alternative formulations.

#### 4.1.2 What does “green” mean anyway?

There is a strong argument that reductive overall red, amber, green scoring can be counterproductive. The “greenest” solvent for a particular application is inherently dependent on the specifics of that case. It is to be hoped that the flexibility of this tool, which allows it to be applied to sub-scores within the various solvent guides allow forthoughtful customisation. The equivalent principle of cautious scepticism should also be extended to claims to as to the coverage (or not) of space by “green” solvents.

#### 4.1.3 How sensitive are the outputs to the parameters chosen?

The *R*_0_ and step size of the solvent ratios can be customised by the user, but in cases where there may be uncertainty as to what these parameters should be set to, one might wonder how dependent the list of solvents suggested for screening might be on the choice of parameters.

As such, for a set solvent ratio step size of 5%, the relative ranking of the top 10 binary solvent mixtures suggested was plotted for *R*_0_ values of 2, 5, 6, 7, 8, 9 and 10 (Fig. 12). Little change can be seen in the relative rankings, suggesting that in many cases any value of *R*_0_ can be used if a simple list of start points for screening is required. It should be noted that for any given binary solvent mixture only the ratio giving the lowest RED was selected. Other ratios may still have lower RED values than other, lower ranked, binary mixtures.

**Fig. 12:**
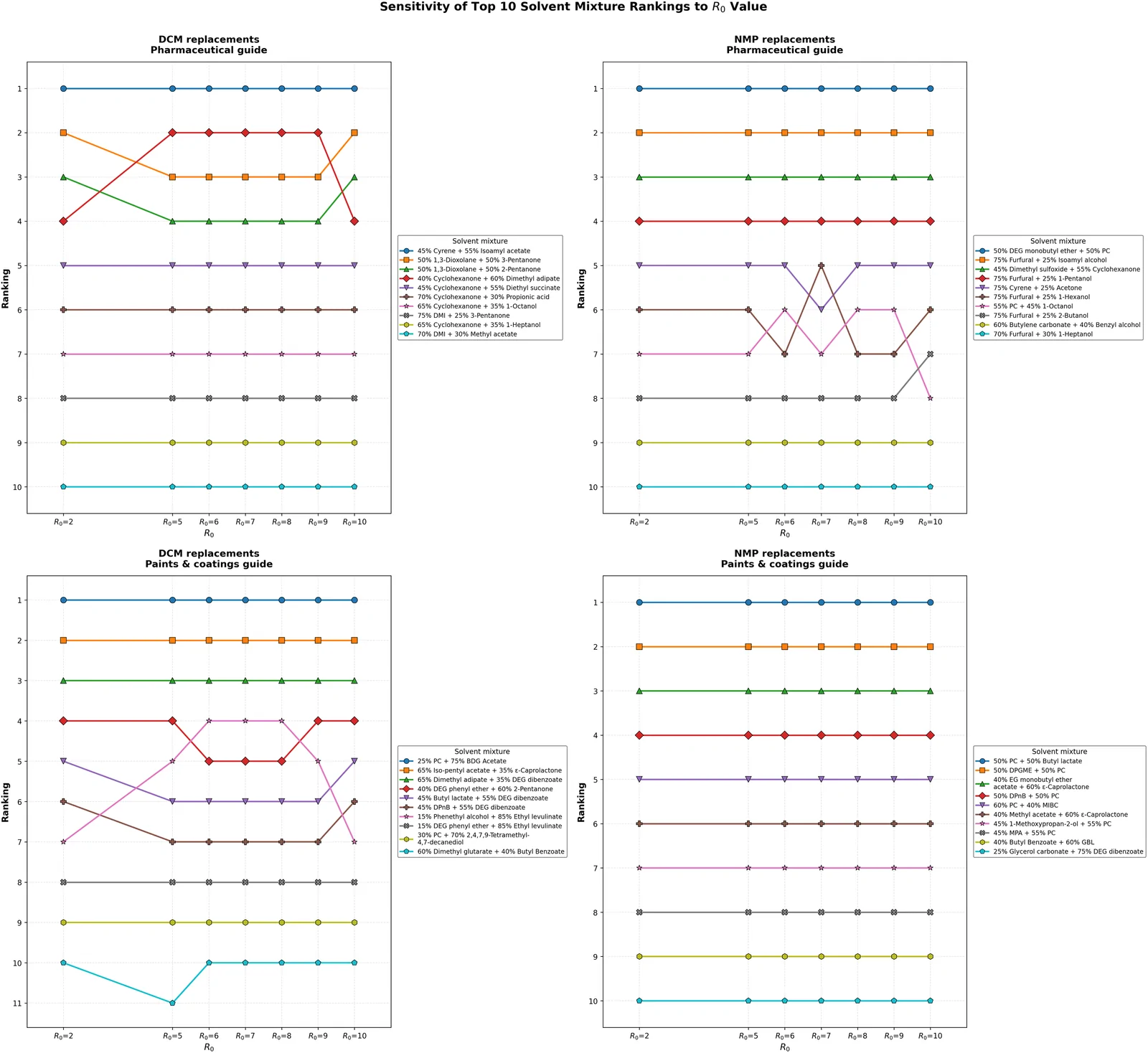
Plot showing the sensitivity of the top 10 ranked solvent mixtures to *R*_0_ from 2 to 10 (using 5% solvent steps). (DMI = dimethyl isosorbide, DEG = diethyleneglycol, PC = propylene carbonate, BDG = butyl diglycol, DPnB = dipropylene glycol, DPGME = dipropylene glycol methyl ether, EG = ethylene glycol, MIBC = methyl iso-butyl carbinol, MPA = propylene glycol methyl ether acetate, GBL = gamma-butyrolactone).

How sensitive the model is to the solvent ratio step size can be judged by the RED values obtained, and as one might expect, using smaller step sizes gives lower RED values in all 4 instances (SI S12).

However, mindful that in many cases solvent properties other that how well the mixture solubilises a given solvent may be critical to the practicality of a suggested ratio, it is reasonable to wonder whether the step size selected changed the identity of the top 10 solvents suggested, or merely the percentage ratios proposed (which could always be optimised from a coarser, 5% step at a later stage).

As such for a set *R*_0_ value of 8, typical of the distance of a marginal solvent from a polymer, the identity of the top 10 binary solvent mixtures (ignoring percentage composition) was plotted for percentage step sizes of 1% and 5% (Fig. 13). The top suggestions were seen to be broadly consistent, suggesting that in many cases the coarser 5% step may be sufficient for a first pass screen prior to further refinement if desired.

**Fig. 13:**
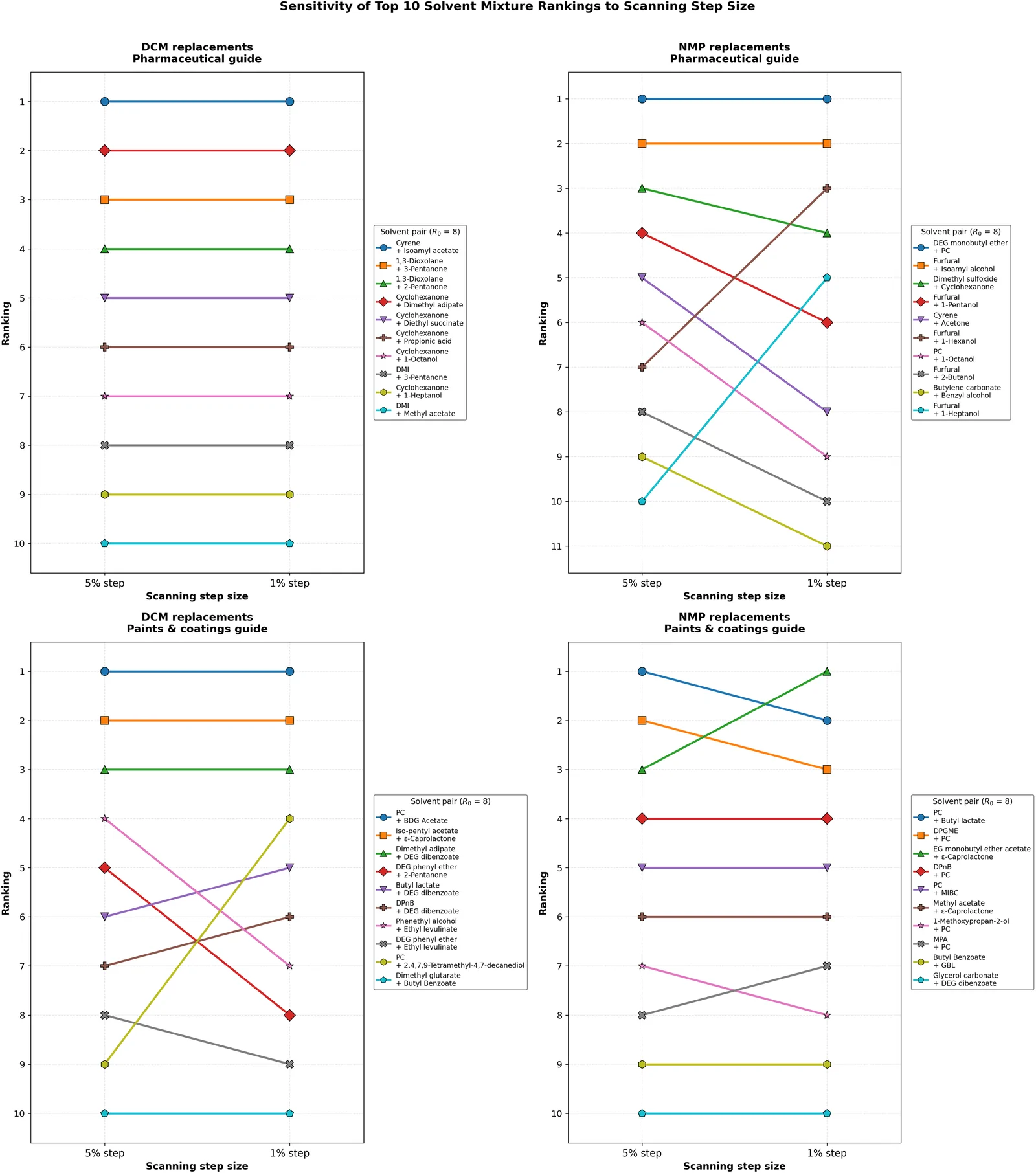
Plot showing the sensitivity of the identity of the top 10 ranked solvent mixtures to % solvent step (using *R*_0_ = 8). (DMI = dimethyl isosorbide, DEG = diethyleneglycol, PC = propylene carbonate, BDG = butyl diglycol, DPnB = dipropylene glycol, DPGME = dipropylene glycol methyl ether, EG = ethylene glycol, MIBC = methyl iso-butyl carbinol, MPA = propylene glycol methyl ether acetate, GBL = gamma-butyrolactone).

### 4.2 Updated consumer and industrial guides

While the updated versions of these guides are largely presented without comment on the understanding that chemists will use these scores, sub-scores, and combinations of scores differently in different contexts and should be free to apply their own judgements, there were some notable observations in the newly scored solvents. Dibasic esters have been widely recommended as “greener” solvents for the paints and coatings industry,^42,43^ and our scoring is consistent with this assessment and continues to give these solvents a green or high Amber overall score. Newly scored dimethyl succinate and dimethyl adipate both scored “green”. Conversely, methyl carbitol, given the commercial availability of biobased ethylene oxide,^44,45^ another (potentially) biobased solvent, was overall graded as “red” by our approach, in large part down to its flammability and reprotoxicity as evidenced by its H361d Globally Harmonised System (GHS) phrase (Suspected of damaging the unborn child).

### 4.3 Comparison of solvent guides

Propylene carbonate was notably observed in 12 of the top 26 recommended potential NMP-replacement solvent mixtures (under the sample conditions chosen) from the pharmaceutical and paint and coating lists (Fig. 7 and 8). Propylene carbonate ranked as amber under both the industrial and consumer paint guides, scoring highly in environmental impact, health impact, and safety. The top ranked NMP-replacement (under the sample conditions chosen) from the paints and coatings list was a propylene carbonate butyl lactate mixture. Butyl lactate ranked overall “green” in both consumer and industrial guides.

Whilst not recommended as frequently, propylene carbonate, in a binary mixture with butyl digylcol acetate, was still the top recommended replacement for DCM from the paints and coatings solvent list (Fig. 6). Butyl diglycol acetate is the second ranked solvent in the consumer guide and the fourth ranked solvent in the industrial guide, exhibiting significantly greener safety, environmental, and health impact scores than DCM. Both solvents in this mixture also have high boiling points, and so would address the major VOC drawbacks associated with DCM.

Interestingly for all values of *R*_0_ explored (see SI) both the pharmaceutical and paints and coatings guides listed more possible alternatives for DCM than for NMP, reflecting the different areas of HSP space that these solvents occupy, but possibly also reflecting the likely ease of substitution of these solvents. This is consistent with previous reviews which have explored alternatives to chlorinated^46^ and dipolar aprotic^47^ solvents and found greener alternatives to chlorinated solvents to be better developed.

### 4.4 Miscibility

The solvent lists used above do not explicitly factor in miscibility of solvents at all. This is intentional, since for many purposes, miscibility of a mixture would not be required for the application, especially if combinations are being used as a start point for further formulation work. For instances where miscibility is required, the reader is directed to miscibility databases and invited to filter their solvent lists, considering the temperature of their required application, before running the software for their purposes. The Merck website^48^ provides a simple table of the miscibilities of 32 common solvents with each other. However a more comprehensive consideration of miscibilities across all solvent ratios may be appropriate for some scenarios, and in those instances the reader is directed to subscription based resources such as DECHEMA DETHERM,^49^ the NIST ThermoData Engine,^50^ Dortmund Data Bank (DDB),^51^ or the CRC Handbook of Chemistry and Physics^52^ amongst others. Or indeed, the HSPs of the component solvents in a mixture can themselves be considered to suggest whether a given mixture is likely to be miscible.

### 4.5 Underserved areas of solvent space

Comparison of the “clouds” of Hansen solubility space covered by either the pharmaceutical solvents list or the paints and coatings list with the positions of the pure solvents in pharmaceutical and paints and coatings, highlights areas of space which are relatively underserved by any solvents, and areas of space which are relatively underserved by greener solvents. This approach complements previous assessments of this space with fewer solvents.^53^

## 5. Conclusions

An extended solvent sustainability guide, including a further 40 solvents, is offered specific to the paints and coatings industry, with scores given for both industrial and consumer applications.

Albrecht and Strube's open access software has been modified to be able to consider red, amber, green overall scores, or sub scores, or other criteria, to facilitate selection of alternative solvents for screening. Diagrams have been developed to help show mixtures of solvents that could be explored to replace certain solvents/solubilising of certain substrates. Excel files and code have been provided (see SI) to facilitate generation of lists, or diagrams, of solvent mixtures postulated as replacement mixtures for any listed solvent, or as possible solvent systems for solubilising a given solute.

The binary solvent mixtures offered as replacements for DCM and NMP in the circle diagrams are an initial snapshot of the greener solutions that can be identified using this toolkit. However, no definitive list of replacements is being proposed here, these are not one-size-fits-all solutions, and care should be taken to customise parameters and scoring in a way that is shaped to users' individual applications. Many different aspects of sustainability can be addressed in this way, quickly providing lists of appropriate solvents with can be investigated further.

The mapping of space, by the binary mixtures highlights areas of space which are relatively underserved by any solvents, not surprisingly, the edges of the cloud, where the outlying solvents of the individual solvent plots feature, and which are relatively underserved by greener solvents. These “deserts” for green solvents include the non-polar low *δ*_P_ region typically served by aliphatic (hexane, white spirit) and aromatic hydrocarbons (toluene, xylenes), the low *δ*_H_ region typically occupied by chlorinated solvents (that the binary mixtures postulated as alternatives to DCM in this paper attempt to address) and the high *δ*_H_, high *δ*_P_ region occupied by dipolar aprotic solvents (that the binary mixtures postulated as alternatives to NMP in this paper attempt to address). It is to be hoped that future research will identify single solvent green alternatives to help fill these gaps, similar to the way tetramethyloxolane has been proposed as an alternative for aromatic hydrocarbons.^31,32^

Whilst outside the scope of this publication, it is also intended for further work to explore the practical validation of this approach for DCM and NMP replacement for a subset of applications.

Longer term it is envisioned that this work could complement existing approaches for solvent selection in the paints and coatings industry,^3,5^ and the pharmaceutical industry^11,54^ by facilitating the consideration of multiple sustainability criteria for different solvent mixtures. Some of these tools already enable filtering by properties, it would be computationally relatively straightforward to also permit filtering by HSP parameters, and to thereby identify lists of binary mixtures for screening meeting additional criteria.

## Conflicts of interest

There are no conflicts to declare.

## Supplementary Material

SU-004-D6SU00271D-s001

SU-004-D6SU00271D-s002

SU-004-D6SU00271D-s003

SU-004-D6SU00271D-s004

SU-004-D6SU00271D-s005

SU-004-D6SU00271D-s006

SU-004-D6SU00271D-s007

SU-004-D6SU00271D-s008

## Data Availability

Python code for the generation of lists of solvents, circle diagrams of various options for screening, and RAG heatmaps in HSP space are openly available on GitHub (https://github.com/helensneddon-arch/solvent-guide-hsp). Supplementary information (SI): consumer and industrial versions of the Paints and Coatings Guide, lists of possible DCM and NMP replacements with variable *R*_0_, RAG heatmaps with variable RED and variable *R*_0_, excel file of the parameters (including scoring). See DOI: https://doi.org/10.1039/d6su00271d.

## References

[cit1] Hansen C. M. , Hansen Solubility Parameters: A User's Handbook, 2nd edn, Taylor & Francis, 2007

[cit2] Enekvist M., Liang X., Zhang X., Dam-Johansen K., Kontogeorgis G. M. (2022). Prog. Org. Coat..

[cit3] Rippatha E., Mendez Rossal H. R., Strauß B., Schwarzinger C. (2025). Clean Technol..

[cit4] https://hansen-solubility.com/HSPiP/green-solvents.php, accessed 03/02/2026

[cit5] Albrecht F., Strube O. I. (2025). J. Coat. Technol. Res..

[cit6] Prat D., Wells A., Hayler J., Sneddon H., McElroy C. R., Abou-Shehada S., Dunn P. J. (2016). Green Chem..

[cit7] Prat D., Wells A., Hayler J., Sneddon H., McElroy C. R., Abou-Shehada S., Dunn P. J. (2015). Green Chem..

[cit8] Alder C. M., Hayler J. D., Henderson R. K., Redman A. M., Shukla L., Shuster L. E., Sneddon H. F. (2016). Green Chem..

[cit9] Alfonsi K., Colberg J., Dunn P. J., Fevig T., Jennings S., Johnson T. A., Kleine H. P., Knight C., Nagy M. A., Perry D. A., Stefaniak M. (2008). Green Chem..

[cit10] Prat D., Pardigon O., Flemming H.-W., Letestu S., Ducandas V., Isnard P., Guntrum E., Senac T., Ruisseau S., Cruciani P., Hosek P. (2013). Org. Process Res. Dev..

[cit11] Diorazio L. J., Hose D. R. J., Adlington N. K. (2016). Org. Process Res. Dev..

[cit12] Stampolaki E., Mondello A., Alladio E., Oliveri P., Mazzoleni A., Pena-Pereira F., Psillakis E. (2026). TrAC, Trends Anal. Chem..

[cit13] Pilon L., Day D., Maslen H., Stevens O. P. J., Carslaw N., Shaw D. R., Sneddon H. F. (2024). Green Chem..

[cit14] European Parliament and Council , Decision No 455/2009/EC Amending Council Directive 76/769/EEC as Regards Restrictions on the Marketing and Use of Dichloromethane, Publications Office of the European Union, Luxembourg City, 2009, L 137, pp. 3–6

[cit15] Barone S. , Selby-Mohamadu Y., Brinkerhoff C. and Koehrn K., TSCA Risk Evaluation for Methylene Chloride (Dichloromethane), Document No. EPA 740-R1-8010, U.S. Environmental Protection Agency, Washington DC, 2020

[cit16] A Guide to Complying with the 2024 Methylene Chloride Regulation under the Toxic Substances Control Act (TSCA) (RIN 2070-AK70), accessed 10th November 2025, DOI: https://www.epa.gov/system/files/documents/2024-07/mecl-compliance-guide.pdf

[cit17] European Chemicals Agency , How to Comply with REACH Restriction 71, Guideline for Users of NMP (1-methyl 2-pyrrolidone), Publications Office of the European Union, Luxembourg City, 2019 https://echa.europa.eu/documents/10162/17233/entry_71_exp_note_biomonitoring_en.pdf/25c684f2-243e-4321-e7c0-ba7074838ab6, accessed 22/02/2026

[cit18] Mansour F. R., Bedair A. (2025). Sustainable Chem. Pharm..

[cit19] Joudan S., Lundgren R. J. (2022). Science.

[cit20] HSP Basics, https://www.stevenabbott.co.uk/practical-solubility/hsp-basics.php, accessed 4/2/2026

[cit21] Surface Tension, Hansen Solubility Parameters, Molar Volume, Enthalpy of Evaporation, and Molecular Weight of Selected Liquids, https://www.accudynetest.com/solubility_table.html, accessed 04/02/2026

[cit22] Vidal M., Rogers W. J., Mannan M. S. (2006). Process Saf. Environ. Prot..

[cit23] Liaw H. J., Lee T. P., Tsai J. S., Hsiao W. H., Chen M. H., Hsu T. T. (2003). J. Loss Prev. Process Ind..

[cit24] Di Benedetto A., Sanchirico R., Di Sarli V. (2018). J. Loss Prev. Process Ind..

[cit25] da Cunha S., Liaw H.-J., Gerbaud V. (2017). Fluid Phase Equilib..

[cit26] Gupta R., Badhe Y., Rai B., Mitragotri S. (2020). RSC Adv..

[cit27] Marren K. (2011). Phys. Sportsmed..

[cit28] Cilurzo F., Vistoli G., Selmin F., Gennari C. G. M., Musazzi U. M., Franzé S., Lo Monte M., Minghetti P. (2014). Mol. Pharm..

[cit29] Occupational Safety and Health Administration Title 29 CFR 1910.1000: Air Contaminants, https://www.osha.gov/laws-regs/regulations/standardnumber/1910/1910.1000, accessed 04/05/2026

[cit30] European Chemicals Agency , Guidance on the Application of the CLP Criteria, Publications Office of the European Union, Luxembourg City, 2024

[cit31] Byrne F., Forier B., Bossaert G., Hoebers C., Farmer T. J., Clark J. H., Hunt A. J. (2017). Green Chem..

[cit32] Link T., Sherwood J., Day D., Kluge M., Farmer T. J., Robert T. (2024). Ind. Eng. Chem. Res..

[cit33] Abbott S. , Hansen C. M. and Yamamoto H., Hansen Solubility Parameters in Practice – Complete with Software, Data and Examples, 5th edn, 2015

[cit34] Steven Abbot solvent blends, https://www.hansen-solubility.com/HSP-science/solvent-blends.php, accessed 09/05/2026

[cit35] Díaz de los Ríos M., Hernández Ramos E. (2020). Discov. Appl. Sci..

[cit36] Evenepoel N., Wen S., Tilahun Tsehaye M., Van der Bruggen B. (2018). J. Appl. Polym. Sci..

[cit37] Rasool M. A., Vankelecom I. F. J. (2019). Green Chem..

[cit38] Dong X., Shannon H. D., Parker C., De Jesus S., Escobar I. C. (2020). AIChE J..

[cit39] Russo F., Galiano F., Pedace F., Aricò F., Figoli A. (2020). ACS Sustain. Chem. Eng..

[cit40] Marino T., Galiano F., Molino A., Figoli A. (2019). J. Membr. Sci..

[cit41] Jailani N. A., Jamil A. H. A., Noh M. H., Moniruzzaman M. (2024). J. Ionic Liq..

[cit42] Kob N. E. , in Clean Solvents: Alternative Media for Chemical Reactions and Processing, ed. M. A. Abraham and L. Moens, American Chemical Society, 2002, ch. 17, vol. 819, pp. 238–253

[cit43] Ismail N., Zhou Q., Wang Q., Cui Z., Skoglund N., Tavajohi N. (2023). Green Chem..

[cit44] Gebers J. C., Marek E. J. (2024). Chem. Eng. J..

[cit45] Stichnothe H. , Bell G., Jørgensen H., De Bari I., Haveren J., Lindorfer J., Kepler J. and de Jong E., Bio-Based Chemicals A 2020 Update Bio-Based Chemicals A 2020 Update with Input from: (Pdf Version) Published by IEA Bioenergy, 2020

[cit46] Jordan A., Stoy P., Sneddon H. F. (2021). Chem. Rev..

[cit47] Jordan A., Hall C. G. J., Thorp L. R., Sneddon H. F. (2022). Chem. Rev..

[cit48] Solvent Miscibility Table, https://www.merckmillipore.com/BA/en/technical-documents/technical-article/analytical-chemistry/purification/solvent-miscibility-table, accessed 04/02/02026

[cit49] DETHERM Database, https://dechema.de/en/detherm.html, accessed 08/2/2026

[cit50] ThermoData Engine, https://www.nist.gov/mml/acmd/trc/thermodata-engine, accessed 08/02/2026

[cit51] DDBST GmbH, https://www.ddbst.com/, accessed 8/2/2026

[cit52] CHEMnetBASE, https://hbcp.chemnetbase.com/contents/ContentsSearch.xhtml?dswid=-9242, accessed 8/2/2026

[cit53] Benazzouz A., Moity L., Pierlot C., Sergent M., Molinier V., Aubry J.-M. (2013). Ind. Eng. Chem. Res..

[cit54] Diorazio L. J., Richardson P., Sneddon H. F., Moores A., Briddell C., Martinez I. (2021). ACS Sustain. Chem. Eng..

